# Effects of altered excitation–inhibition imbalance by repetitive transcranial magnetic stimulation for self-limited epilepsy with centrotemporal spikes

**DOI:** 10.3389/fneur.2023.1164082

**Published:** 2023-05-26

**Authors:** Yujiao Yang, Yixian Han, Jing Wang, Yongkang Zhou, Dong Chen, Mengyang Wang, Tianfu Li

**Affiliations:** ^1^Department of Neurology, Sanbo Brain Hospital, Capital Medical University, Beijing, China; ^2^CAS Key Laboratory of Mental Health, Institute of Psychology, Beijing, China; ^3^Beijing Key Laboratory of Epilepsy, Sanbo Brain Hospital, Capital Medical University, Beijing, China; ^4^Beijing Institute for Brain Disorders, Capital Medical University, Beijing, China

**Keywords:** self-limited epilepsy with centrotemporal spikes, repetitive transcranial magnetic stimulation, excitation-inhibition imbalance, spike-wave index, electrical status epilepticus in sleep

## Abstract

**Objectives:**

Patients with self-limited epilepsy with centrotemporal spikes (SeLECTS) with electrical status epilepticus in sleep (ESES) have generalized cognitive impairment, yet treatment options are limited. Our study aimed to examine the therapeutic effects of repetitive transcranial magnetic stimulation (rTMS) on SeLECTS with ESES. In addition, we applied electroencephalography (EEG) aperiodic components (offset and slope) to investigate the improvement of rTMS on the excitation–inhibition imbalance (E-I imbalance) in the brain of this group of children.

**Methods:**

Eight SeLECTS patients with ESES were included in this study. Low-frequency rTMS (≤1 Hz) was applied for 10 weekdays in each patient. To assess the clinical efficacy and changes in E-I imbalance, EEG recordings were performed both before and after rTMS. Seizure-reduction rate and spike-wave index (SWI) were measured to investigate the clinical effects of rTMS. The aperiodic offset and slope were calculated to explore the effect of rTMS on E-I imbalance.

**Results:**

Five of the eight patients (62.5%) were seizure-free within 3 months after stimulation, with treatment effects decreasing with longer follow-ups. The SWI decreased significantly at 3 and 6 months after rTMS compared with the baseline (*P* = 0.0157 and *P* = 0.0060, respectively). The offset and slope were compared before rTMS and within 3 months after stimulation. The results showed a significant reduction in the offset after stimulation (*P* < 0.0001). There was a remarkable increase in slope after the stimulation (*P* < 0.0001).

**Conclusion:**

Patients achieved favorable outcomes in the first 3 months after rTMS. The ameliorative effect of rTMS on SWI may last up to 6 months. Low-frequency rTMS could reduce firing rates in neuronal populations throughout the brain, which was most pronounced at the site of stimulation. A significant reduction in the slope after rTMS treatment suggested an improvement in the E-I imbalance in the SeLECTS.

## 1. Introduction

Self-limited epilepsy with centrotemporal spikes (SeLECTS) is the most common focal syndrome in childhood epilepsy (1). Most children with SeLECTS have a good prognosis, but a small percentage may evolve into epileptic encephalopathy with spike-and-wave activation in sleep (EE-SWAS). The EEG pattern associated with EE-SWAS is known as electrical status epilepticus in sleep (ESES) (2). The nearly constant epileptiform activity of slow-wave sleep is usually accompanied by significant regression in cognitive or behavioral function. All cognitive domains may be affected, including language and communication, temporospatial orientation, attention, and social interaction. However, existing treatments remain very limited in their ability to effectively reduce functional impairment in SeLECTS patients with ESES.

Repetitive transcranial magnetic stimulation (rTMS), as a focal, non-invasive technique, has therapeutic potential in the field of epilepsy (3). Low-frequency rTMS (≤1 Hz) inhibits cortical excitability, increases cortical silent period duration, and reduces motor-evoked potential amplitudes (4). The rationale for using low-frequency rTMS to suppress seizures is related to the fact that it is promising to interrupt synaptic potential and focal cortical excitability. Real-world evidence suggests that low-frequency rTMS using a figure-8-coil may be an effective therapy for drug-resistant epilepsy in pediatric patients, resulting in a 30% reduction in seizure frequency (5). Ren et al. found that rTMS acted as a novel approach to behavioral problems that are highly prevalent in patients with SeLECTS (6). Although a Cochrane review found rTMS to be safe and effective in reducing epileptiform discharges, the evidence for the efficacy of rTMS for seizure reduction is still lacking (7).

The imbalance between excitatory and inhibitory properties (E-I imbalance) in SeLECTS has been identified as contributing to seizures and cognitive impairment (8). The inhibitory network involves both sensorimotor and subcortical networks, which manifest as a dissociation of the corresponding functions. However, the effect of rTMS on improving the E-I imbalance in SeLECTS patients is unclear. We hypothesized that rTMS would reduce seizure frequency and E-I imbalance in SeLECTS. To address our hypothesis, two requirements need to be met: (1) whether seizure frequency and epileptiform discharges are reduced after rTMS and (2) whether the E-I imbalance can be improved by rTMS.

## 2. Methods

### 2.1. Patient selection

This study was a retrospective analysis of patients with SeLECTS who visited the Sanbo Brain Hospital from January 2015 to December 2020. The inclusion criteria were as follows: (1) age of onset between 3 and 15 years; (2) appropriate seizure semiology which suggested focal onset (9); (3) centrotemporal spikes on EEG and unilateral spike-wave index (SWI) >80% or bilateral SWI >50%; (4) the neuropsychology test showed that intelligence quotient (IQ) was lower than normal intellectual development; (5) TMS treatment; and (6) at least one EEG exanimation after TMS. We excluded children with a history of prematurity (<35 weeks), abnormal magnetic resonance imaging (MRI) of the brain, other epilepsy syndromes, neurosurgery, and severe brain injury. This study was approved by the Ethics Committee of Sanbo Brain Hospital, Capital Medical University.

### 2.2. EEG recordings

The EEGs were recorded based on the standard international 10–20 system, with 19 scalp electrodes. The recording system used was Nicolet. EEG recordings during sleep and awake were obtained. The EEG signal was recorded at 512 Hz or 1,024 Hz. Impedance levels for each electrode were at or below 50 kΩ during data collection. EEG was referenced online to the central midline electrode site (Cz). The SWI was reviewed by an independent epileptologist blinded to the clinical data.

### 2.3. Neuropsychological evaluation

All patients underwent evaluation for intellectual and behavioral impairment using standard assessment procedures. The Wechsler Intelligence Scale for Children, fourth edition (WISC-IV), was used to measure the intelligence level of children at the baseline.

### 2.4. rTMS procedure

In our study, rTMS was performed on all patients using Magstim (Company Ltd.). The figure-8-coil plane targeting the stimulation site was tangential and was kept parallel to the scalp. The stimulus parameters were as follows: frequency ≤1 Hz; intensity, reference resting motor threshold; and the number of stimuli, 500/1,000/1,500 per site, depending on the frequency. The treatment lasted for 10 weekdays. The stimulation site was the central region (C5 or C6). The determination of the stimulation site was based on a combination of seizure symptoms, EEG, and abnormalities of positron emission tomography (PET) metabolism in the brain.

### 2.5. EEG data analysis

The EEGLAB toolbox in MATLAB was used to analyze the raw EEG data. The EEG was re-referenced to the average of all electrodes. We applied a 1 Hz bandpass filter and 80 Hz cutoff to the data. Independent components analysis was used to correct eye blink artifacts for correction.

The power spectral density (PSD) was calculated at 0.5 Hz increments from 1 Hz to 40 Hz using Welch's method (10 s time window, 0.5 s window length, 50% overlap). The “Fitting Oscillations and One-Over-f” (FOOOF) toolbox was used to calculate the aperiodic component (offset and slope). The PSD slope is equivalent to the negative exponent when measured in log–log space due to aperiodic activity having a 1/f-like distribution with exponentially decreasing power across increasing frequencies (10). The E-I ratio could be estimated from the PSD slope. The steeper the slope is, the lower the E-I ratio (11). A more negative slope indicates that relatively more inhibition occurs in the underlying neuronal populations (12). The power spectrum, P, was modeled using three parameters:


(1) $$\begin{array}{l} P=L+\overset{N}{\underset{n=0}{\sum }}G_{n}\;, \end{array}$$

where *L* is the aperiodic “background” signal, with *N* total peaks extracted from the power spectrum and Gaussians (*Gn*) fitted to each peak. The peaks were iteratively fitted by Gaussians:


(2) $$\begin{array}{l} G_{n}=a*\exp\left(\frac{-{\left(F-c\right)}^{2}}{2w^{2}}\right), \end{array}$$

with *an* amplitude, center frequency, *c*, the bandwidth, *w*, of the Gaussian *G*, and the input frequencies, *F*. The aperiodic signal *L* was modeled by


(3) $$\begin{array}{l} L=b-\log\left(k+F^{\chi }\right), \end{array}$$

where *b* is the broadband offset, *x* is the slope, and *k* is the “knee” parameter, which was set to 0. The FOOOF model was fitted for the frequency range of 1–40 Hz.

### 2.6. Follow-up program

Each patient was assessed for seizure frequency, SWI, WISC-IV, offset, and slope before treatment and observed after 10 working days of low-frequency stimulation. Seizure frequency, SWI, offset, and slope were assessed at 3 months post-treatment. Seizure frequency and SWI were measured at 6 months and 12 months post-treatment.

### 2.7. Statistical analysis

To test whether there were significant differences between baseline measurements before rTMS and follow-up measurements after rTMS, the pairwise comparison method was applied. First, a normality test was performed. If all groups met the normality and the variation between the two groups was homogeneous, a paired *t*-test was used. A non-parametric Wilcoxon matched-pairs signed rank test was considered if all groups did not meet the normality test. *P*-values < 0.05 were considered to be statistically significant. *P-*values were corrected with the original false discovery rate (FDR) method of Benjamini and Hochberg.

## 3. Results

### 3.1. Clinical information

Eight patients with SeLECTS were enrolled, 4 female and 4 male patients. Follow-up visits were scheduled at 3, 6, and 12 months after rTMS. The follow-up schedule is shown in Supplementary Figure S1. The mean age at the onset of epilepsy was 4.5 years (range 3–7), and the mean age at the first visit was 5.9 years (range 4–7). Two children (patients 2 and 4) had a previous history of febrile convulsions. The mean SWI before rTMS was 86.58% (range 74.33–97.67%). MRI was normal in all of them. The mean IQ was 76.5 (range 67–84). Detailed information is listed in Table 1 and Supplementary Tables S1, S2.

**Table 1:** Patients' clinical information.

**Pt/No**.	**Gender**	**Epilepsy onset**	**Age at rTMS**	**Previous history**	**Frequency (Seizures per month)**	**SWI before rTMS (%)**	**MRI**	**ASMs**
1	Female	5	7	Normal	2	97.67	Normal	ZNS, CZP, steroids
2	Female	5	7	Febrile convulsion	0.5	75.33	Normal	VPA, LEV, CZP, steroids
3	Male	5	5	Normal	30	90	Normal	VPA, TPM
4	Female	4	7	Febrile convulsion	30	74.33	Normal	VPA, LEV, steroids
5	Male	4	5	Normal	2	91.33	Normal	LCM, TPM, OXC
6	Male	3	5	Normal	3	94.67	Normal	VPA, LEV, CLB, steroids
7	Male	3	4	Normal	4	82	Normal	VPA, LEV, CZP
8	Female	7	7	Normal	2	87.33	Normal	VPA, LEV

ASMs, anti-seizure medications; ZNS, zonisamide; CZP, clonazepam; VPA, valproate; LEV, levetiracetam; TPM, topiramate; LCM, lacosamide; OXC, oxcarbazepine; CLB, clobazam.

### 3.2. Treatment effects after rTMS

#### 3.2.1. Seizure frequency reduced by rTMS

**Figure 1:**
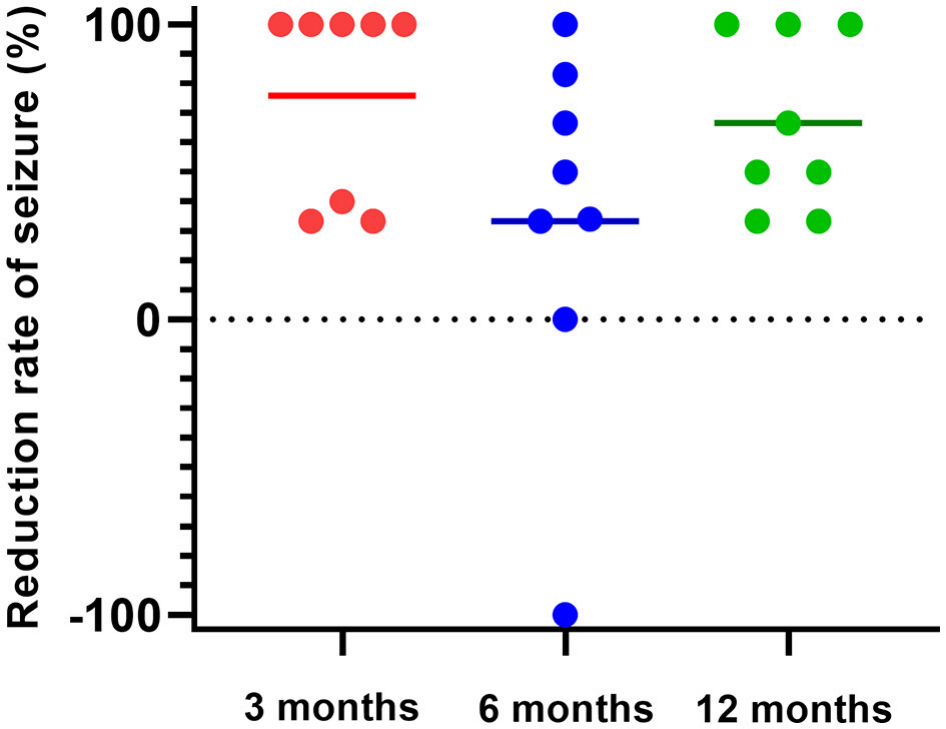
Seizure frequency reduced by rTMS. Five of the eight patients (62.5%) were seizure-free within 3 months after stimulation, and the other three had less than a 50% reduction in seizure frequency (red). Within 6 months after rTMS, seizure frequency was reduced by more than 50% in four patients, and approximately 30% in two patients, returned to the baseline level in one patient, and increased in one patient (blue). Within 12 months after rTMS, three patients had achieved complete seizure-free, three had seizure reduction by more than 50%, and two decreased by approximately 30% (green).

Five of the eight patients (62.5%) were seizure-free within 3 months after stimulation, and the other three had < 50% reduction in seizure frequency. Within 6 months after rTMS, four patients had a reduction in seizure frequency of more than 50%, two patients had a reduction of about 30%, one patient returned to baseline and one patient had an increase in seizure frequency. Within 12 months after rTMS, three patients had achieved complete seizure-free status, three had seizure reduction by more than 50%, and two had a decrease by approximately 30% (Figure 1).

#### 3.2.2. SWI before and after rTMS

**Figure 2:**
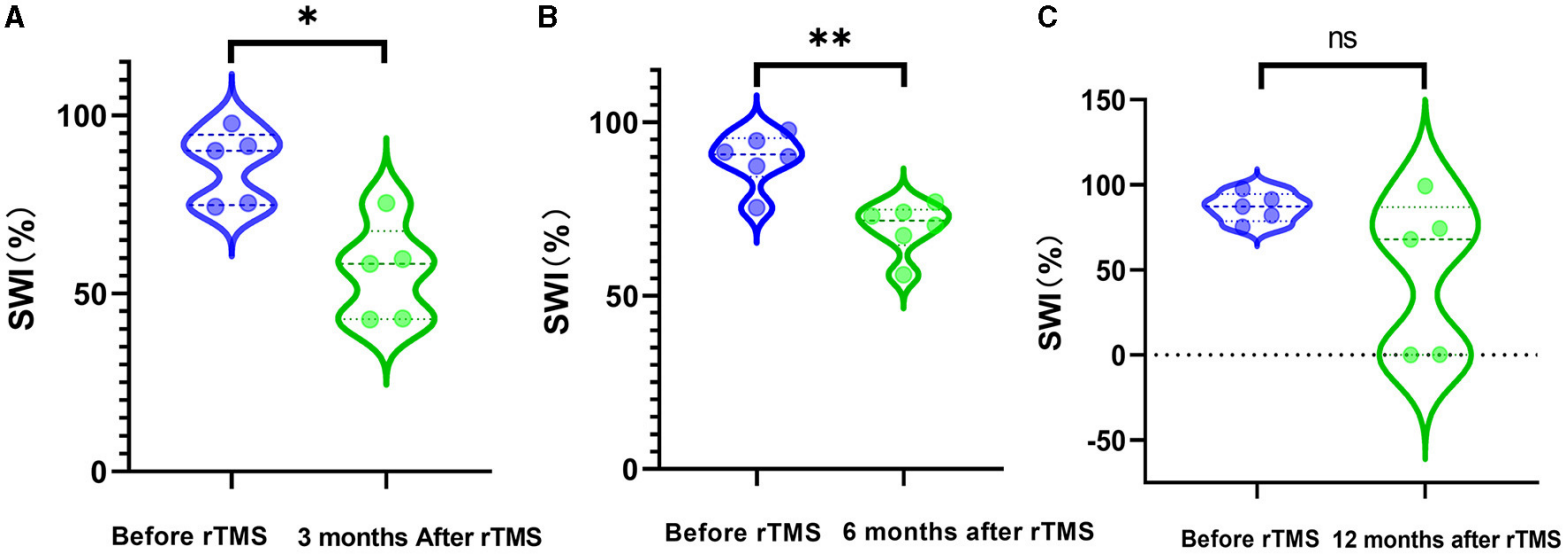
SWI before and after rTMS. **(A)** SWI before rTMS and 3 months after rTMS. **(B)** SWI before rTMS and 6 months after rTMS. **(C)** SWI before rTMS and 12 months after rTMS.

The SWI at 3 months was reduced significantly by rTMS (*P* = 0.0157) (Figure 2A). The SWI at 6 months after rTMS was remarkedly lower than that before rTMS (*P* = 0.0060) (Figure 2B). There was no significant difference in SWI at 12 months after rTMS and before rTMS (Figure 2C).

### 3.3. Evolution of excitability after rTMS

#### 3.3.1. Aperiodic offset before and after rTMS

**Figure 3:**
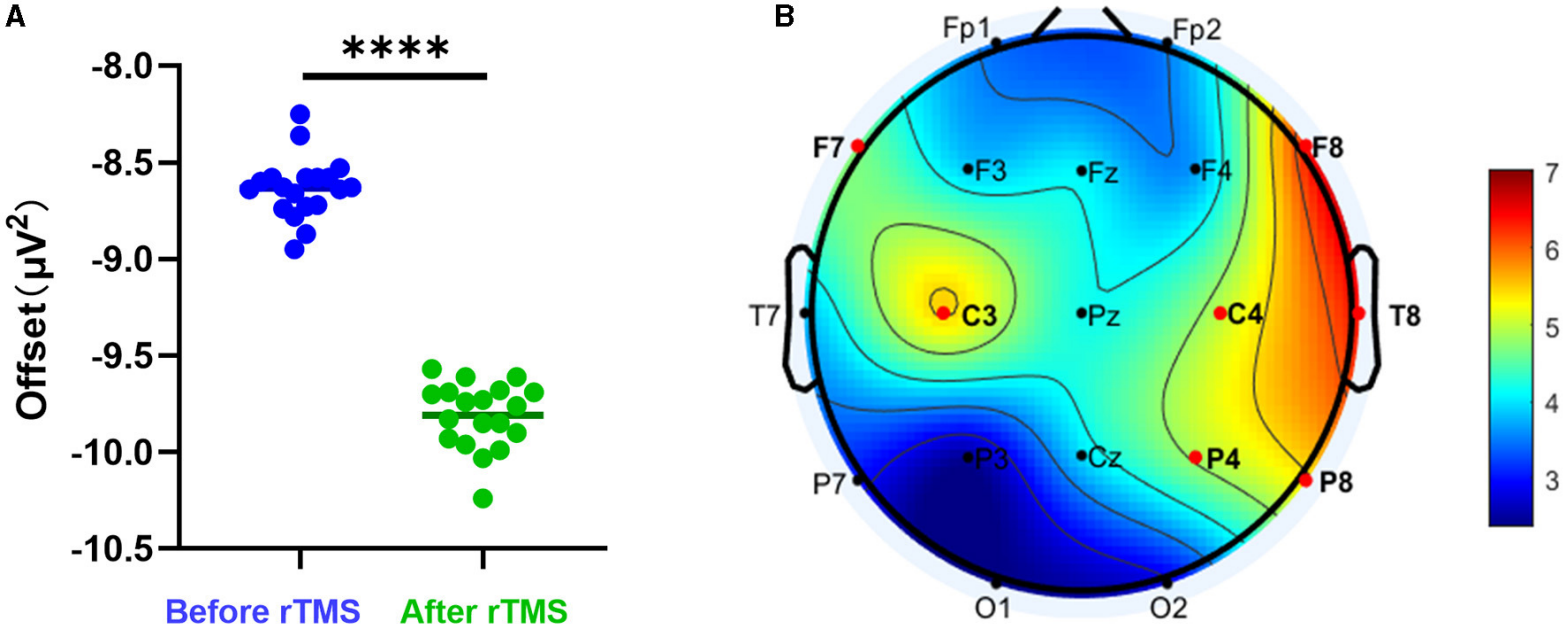
Aperiodic offset before and after rTMS. **(A)** PSD offset before rTMS and 3 months after rTMS. There was a significant reduction in PSD offset after stimulation (*P* < 0.0001). **(B)** T-value distributed in different brain regions.

We evaluated whether rTMS could alter the PSD offset. The offset of all scalp electrodes before rTMS was compared with that at 3 months after rTMS. A significant decrease was observed after stimulation (*P* < 0.0001) (Figure 3A). We further analyzed whether differences existed in different brain regions. It was found that the most significant changes were in brain areas around the stimulation sites (Figure 3B).

#### 3.3.2. Aperiodic slope before and after rTMS

**Figure 4:**
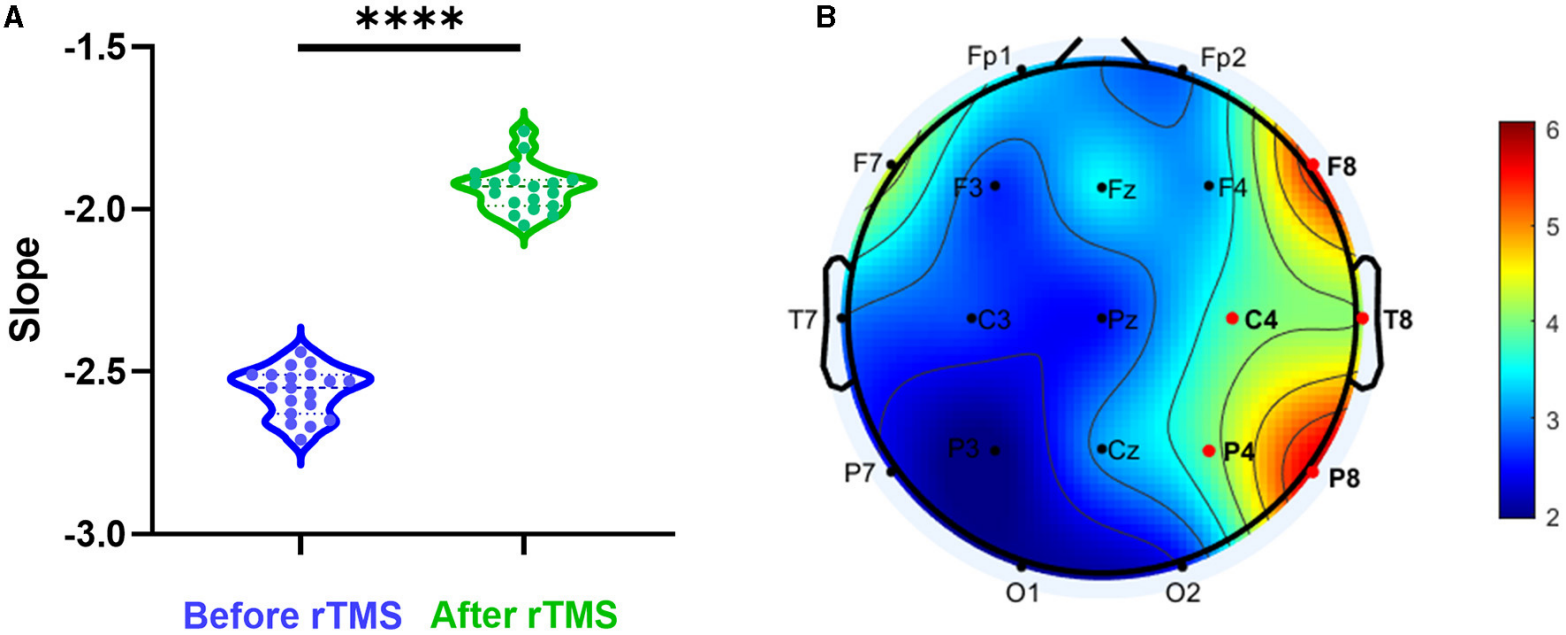
Aperiodic slope before and after rTMS. **(A)** PSD slope before rTMS and 3 months after rTMS. There was a significant reduction in PSD slope after stimulation (*P* < 0.0001). **(B)** T-value distributed in different brain regions.

We assessed whether rTMS could change the E-I imbalance. The PSD slope of all scalp electrodes before rTMS and 3 months after rTMS was compared. It was found that there was a significant increase in PSD slope after stimulation (*P* < 0.0001) (Figure 4A). To analyze the differences in different brain regions, we observed that the most prominent alteration was in the scalp electrodes at the stimulation site (Figure 4B).

## 4. Discussion

The present study aimed to evaluate whether rTMS could clinically benefit SeLECTS patients and modify E-I imbalance. To achieve this goal, our research was divided into two parts. In the first part, we assessed the clinical efficacy of TMS. Seizure-reduction rate and SWI were compared before and after rTMS. The results found that rTMS reduced seizure frequency for at least 3 months. The SWI was decreased by rTMS at 3 and 6 months. In the second part, we applied a special parametric approach to analyze the aperiodic offset and slope. We observed a significant reduction in aperiodic offset after rTMS, reflecting a decline in the spiking rate of cortical neurons. Among all channels, the most dramatic changes occurred around the rTMS stimulation site. The aperiodic slope after rTMS was increased, suggesting that the E-I imbalance was altered by stimulation. Similarly, the most obvious change was around the rTMS site.

### 4.1. Excitation–inhibition imbalance in SeLECTS

SeLECTS is characterized cardinally by sensory-motor seizures, oro-pharyngo-laryngeal symptoms, speech arrest, and hypersalivation, which are associated with abnormal discharges in the Rolandic areas (13, 14). Children with SeLECTS have a less stable network of areas involved in sensorimotor function (15). The function of bilateral sensorimotor areas is disorganized, as evidenced by a significant delay in motor control and impaired language function (16). The diseased neuronal networks are less efficient and may cause language impairments (17). The deficits in the language domain may be a downstream effect of altered motor cortex function possibly due to diffusion of activity to areas involved in language processing and/or as a result of motor execution difficulties, such as tongue immobility (17–19).

The concept of epilepsy as a spectrum disorder is increasingly acknowledged and patients frequently exhibit comorbid cognitive and behavioral impairments (20). Patients with SeLECTS with ESES exhibit an onset of symptoms during a critical period of brain development, which is the most vulnerable time for cognitive function. In addition to seizures, this group of patients is often associated with reduced cognitive function and impaired executive function (21, 22). Of the numerous factors that influence executive dysfunction in patients with SeLECTS, the most significant correlations are observed with the age of onset, frequency of intermittent discharges, and alterations in brain networks (22). During early childhood, the brain exhibits high levels of neuroplasticity and relatively low functional specificity of neural networks. Seizures at this stage can significantly impact children's executive and memory functions (23). The effect on the child's attentional network varies with the frequency of intermittent discharges and is more pronounced if the onset occurs at a younger age of onset (24). Functional and structural brain connections may be altered in children with SeLECTS, resulting in cognitive dysfunction, particularly executive function abnormalities (25, 26). Relevant evidence derived from resting-state functional MRI also suggests that reduced functional connectivity in Rolandic areas may exert an impact on the wider brain network (27).

Numerous studies have shown that the balance between excitatory and inhibitory electrical activity of neurons in the brain is dynamically regulated under normal conditions. However, in patients with epilepsy, the balance is disturbed, resulting in a relative increase in excitatory neuronal activity, either directly or indirectly (28, 29). Studies based on support vector machine models of Granger causal density have revealed abnormalities in connectivity both between and within different networks in patients with SeLECTS (30). Frequent intermittent epileptic discharges can lead to irreversible reconfiguration of neural networks, resulting in an imbalance between excitation and inhibition (31). The excitation–inhibition imbalance in brain networks further leads to cognitive dysfunction and seizures (20, 32), making it a potential biological marker for SeLECTS (8).

### 4.2. RTMS improves E-I imbalance in SeLECTS

TMS exhibits diagnostic and therapeutic potential in the field of epilepsy. As an assessment tool, TMS can be combined with EMG to provide biological markers for indicators of cortical excitation and inhibition associated with epilepsy and antiepileptic drugs (33). Therapeutically, low-frequency rTMS (≤1 Hz) can effectively prolong postsynaptic inhibition and reduce brain excitability. During both interictal and ictal periods, TMS can be utilized to varying degrees to reduce the frequency or severity of seizures or even terminate them (3). However, few studies are using EEG signals to evaluate the improvement of the cortical excitation–inhibition ratio by TMS. The present study may serve as a supplementary and guiding reference for rTMS treatment in epilepsy.

Studies of intracranial local field potential have shown that broadband power offsets are positively correlated with the firing rate of neuronal populations (34). Similar results have been observed in macaque studies correlating neuronal action potentials with instantaneous changes in broadband local field potentials (35). Other studies have shown that whole-brain aperiodic offset is inversely correlated with age, and it is speculated that this phenomenon may be due to a decrease in the firing rate of cortical neurons as the brain matures with age (36). In the present study, the PSD offset decreased after rTMS, reflecting the reduction in cortical neuronal firing and the suppression of action potentials in neuronal populations. Although this was observed on all electrodes, it tended to be more pronounced near the stimulated area. However, the explanatory relationship between the observed phenomenon and the mechanism of TMS remains speculative, and a more precise causal relationship requires further experimental confirmation.

The excitation–inhibition imbalance may lead to hyper-synchronization of the electrical activity of neurons in epileptic networks (29). The slope of the aperiodic PSD signal reflects the balance between excitation and inhibition (11). With an increase in age, the aperiodic slope tends to decrease. In studies of visual working memory tasks, older adults show a relatively flat slope, whereas younger adults show a steeper slope (37). Relevant evidence suggests that an increase in excitation–inhibition ratio may result in a flatter PSD slope, indicating a reduced synchronization of neuronal firing (38). A positive correlation was found between the excitation–inhibition ratio and the PSD slope, while a stronger correlation was found between the synaptic density of inhibitory neurons and the PSD slope. In the present study, the increase in PSD slope after rTMS treatment might be due to the increased application of stimulation effects to inhibitory neurons, resulting in improved function of the SeLECTS inhibitory loop and, thus, a reduction in the excitation–inhibition imbalance. However, this is conjecture, and further studies are needed to elucidate the cytological and molecular mechanisms involved.

## 5. Limitations

There are some limitations in the present study. The sample size was 8, and there was some individual variation that may have influenced the results. The number of EEG electrodes was 19, which may have led to a lack of accuracy in the spatial sampling of electrical brain activity. We did not assess the Wechsler Intelligence Scale for post-treatment assessment and thus cannot yet determine whether rTMS can improve cognitive performance. The most important and difficult point is that since the study is limited to the processing of EEG signals, the results are presented as phenomena and we are not yet able to explain the mechanism of action of rTMS. We have established a more standardized process to compensate for the above limitations to allow for more discoveries in the future.

## 6. Conclusion

Favorable clinical outcomes are observed in patients within the initial 3-month period following rTMS treatment. After treatment, the patient's SWI is significantly reduced, and the effect lasts for up to 6 months. Low-frequency rTMS induces a reduction in neuronal firing, particularly at the site of stimulation. The E-I imbalance can be improved in SeLECTS after rTMS intervention.

## Data availability statement

The raw data supporting the conclusions of this article will be made available by the authors, without undue reservation.

## Ethics statement

The studies involving human participants were reviewed and approved by Ethics Committee of Sanbo Brain Hospital, Capital Medical University. Written informed consent to participate in this study was provided by the participants' legal guardian/next of kin.

## Author contributions

TL and MW contributed to the conception and design of the study. YY and YH contributed to the writing and preparation of the figures. YH and YZ contributed to analyzing the data. JW contributed to reviewing the EEG data. DC contributed to interpreting the results. All authors contributed to the acquisition and analysis of data, reviewed, and revised the manuscript for intellectual content.
