# Supplementary material for: Effects of altered excitation–inhibition imbalance by repetitive transcranial magnetic stimulation for self-limited epilepsy with centrotemporal spikes
Source: Front Neurol. 2023 May 26;14:1164082. doi: 10.3389/fneur.2023.1164082 (PMC10250617; doi:10.3389/fneur.2023.1164082)

Supplementary material

Table S1 Patient's symptomatology information

| Pt/No. | Seizure type | | Developmental disorders |
| --- | --- | --- | --- |
|  | Motor onset | Non-Motor |  |
| 1 | left facial/ hemibody tonic-clonic; | atypical absence;eyelid myoclonus;hypersalivation | intellectual disability; motor regression (Oropharyngeal dystonia); speech disorder |
| 2 | unilateral facial/ hemibody tonic-clonic |  | inattention |
| 3 | Negative myoclonus |  | inattention |
| 4 | eyelid myoclonus; | atypical absence;hypersalivation | learning difficulty |
| 5 | unilateral facial/ hemibody tonic-clonic |  | inattention |
| 6 | unilateral facial/ hemibody tonic-clonic | atypical absence | motor regression (Oropharyngeal dystonia); speech disorder; disturbances of memory |
| 7 | unilateral facial/ hemibody tonic-clonic |  | intellectual disability |
| 8 | unilateral facial/ hemibody tonic-clonic;GTCS | eyelid myoclonus | impaired ability to calculate |

Table S2. Patients’ stimulus parameters of rTMS

| Pt/No. | rTMS | | | |  | Reasons for stimulation site | | |
| --- | --- | --- | --- | --- | --- | --- | --- | --- |
|  | Site | Intensity | Frequency | Number of stimuli |  | symptom | EEG discharge | PET |

| 1 | C6 | 60% MT | 0.5 Hz | 1000 |  | left facial clonic | Bilateral | Not done |
| --- | --- | --- | --- | --- | --- | --- | --- | --- |
| 2 | C5 | 50% MT | 0.5 Hz | 1000 |  | weak in lateralization | Bilateral | Hyperperfusion around C5 |
| 3 | C6 | 40% MT | 0.33Hz | 1500 |  | weak in lateralization | Bilateral; more prominent on the right side | Not done |
| 4 | C6 | 40% MT | 0.33Hz | 1500 |  | weak in lateralization | Bilateral; more prominent on the right side | Not done |
| 5 | C6 | 50% MT | 0.5 Hz | 1000 |  | weak in lateralization | Bilateral; more prominent on the right side | Not done |
| 6 | C6 | 40% MT | 1 Hz | 500 |  | weak in lateralization | Bilateral; more prominent on the right side | Hyperperfusion around C6 |
| 7 | C6 | 40% MT | 1 Hz | 500 |  | weak in lateralization | Bilateral; more prominent on the right side | Hypoperfusion around C6 |
| 8 | C6 | 50% MT | 0.5 Hz | 1000 |  | weak in lateralization | Bilateral; more prominent on the right side | Not done |

**Fig S1 Follow-up program**


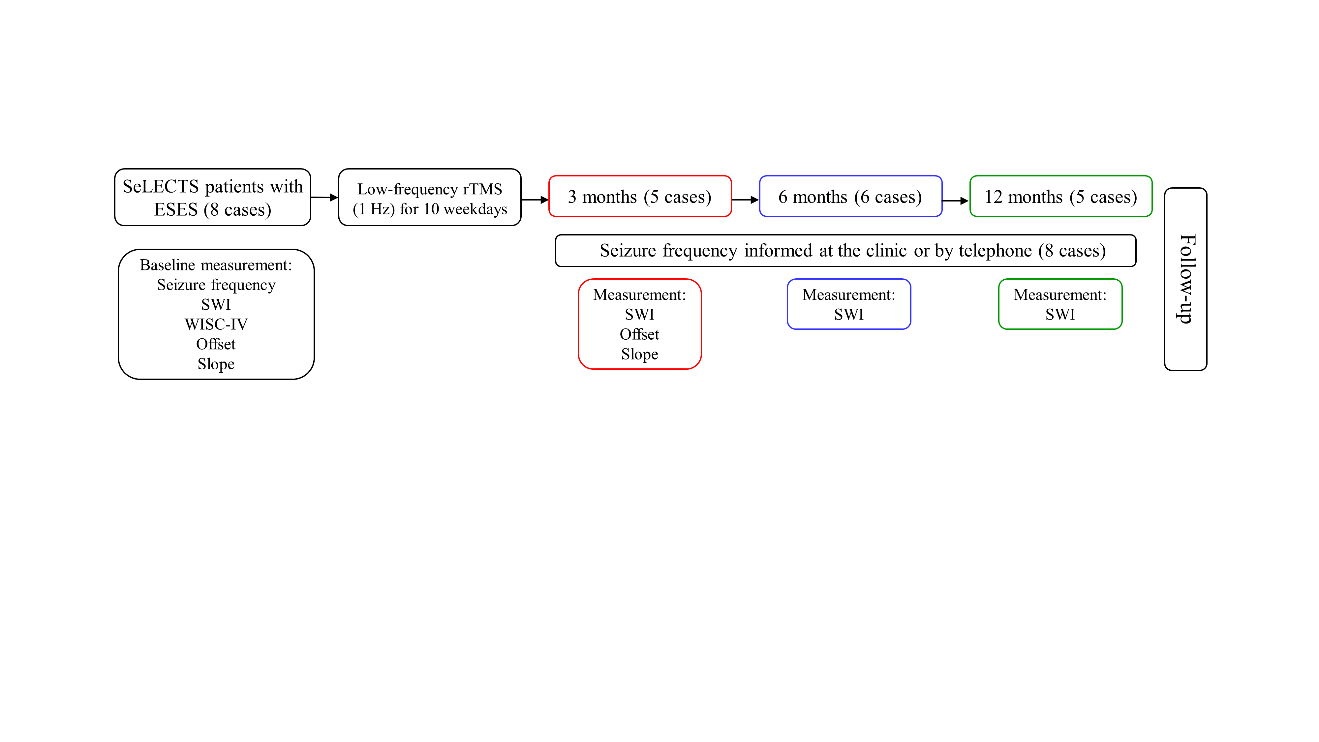


Follow-ups were scheduled at 3, 6, and 12 months after rTMS.

**Fig S2 Individual data for each patient**


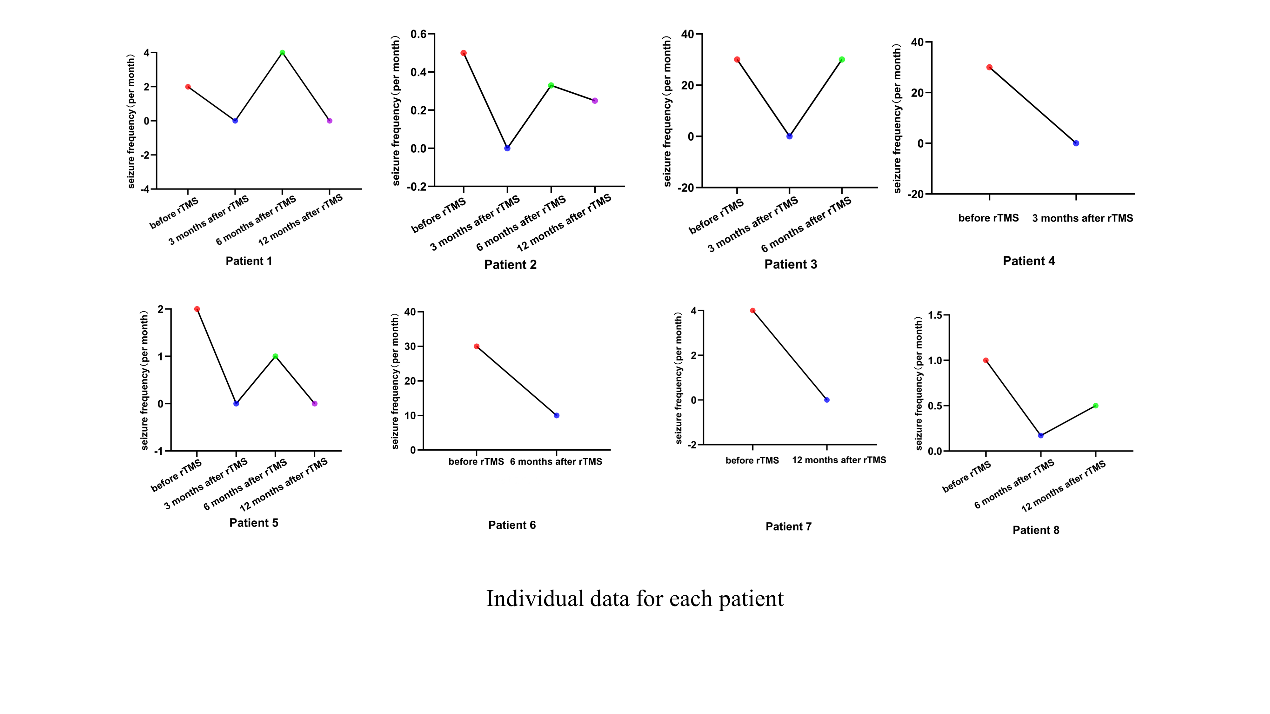

Supplement: Supplementary file 1 [file Data_Sheet_1.docx]
